# Development and content evaluation of a multidimensional coping model-based health education manual for dementia care support

**DOI:** 10.3389/fpubh.2026.1750034

**Published:** 2026-03-16

**Authors:** Jie Wu, Dandan Zhang, Ling Feng, Qinghua Zhao, Mingzhao Xiao, Jun Wang

**Affiliations:** 1 Department of Nursing, The First Affiliated Hospital of Chongqing Medical University, Chongqing, China; 2 Qinggang Senior Care Center, The First Affiliated Hospital of Chongqing Medical University, Chongqing, China

**Keywords:** dementia, dementia care support, health education, manual, multidimensional coping strategies

## Abstract

**Objective:**

This study aimed to develop an evidence- and theory-based health education manual for dementia care and to test its validity and reliability.

**Methods:**

A multi-phase study design was employed to develop a health education manual for caregivers of persons with dementia (PWD). A systematic review of randomized controlled trials identified effective interventions, while parallel semi-structured interviews with caregivers explored practical needs. Then, the initial content framework of the manual was developed based on the previously established Dementia Caregiver Stress Process Model (DeCare-SPM), which informed the organization of content around three core coping strategies: problem-focused, emotion-focused, and meaning-focused coping. Based on this framework, a multidisciplinary team refined and translated the material into accessible, practice-oriented guidance through scientific adaptation and collaborative design. The manual was finalized through expert validation using the Delphi method.

**Results:**

A total of 31 studies were included in the systematic review, with psychosocial and psychoeducational approaches identified as the most common across seven intervention categories. Family caregivers of PWD faced social support barriers and needed dementia knowledge, skills, and emotional stress management. A content system for the education manual was established, comprising three domains (i.e., problem-focused, emotion-focused, and meaning-focused coping), nine secondary indicators, and thirty-one tertiary indicators. The problem-focused domain covered disease knowledge, daily care, and symptom management; the emotion-focused domain included stress adaptation, mindfulness training, and behavioral activation, and the meaning-focused domain focused on self-management and psychological adjustment. The manual incorporated scientifically designed illustrations and multimedia resources (QR codes), and expert evaluation demonstrated high content validity (CVI = 0.81–1.00).

**Conclusion:**

This study developed a health education manual for caregivers of PWD, grounded in caregivers’ needs and evidence-based practices, structured around a multidimensional coping model, and delivered in both paper and digital formats with identical content. Expert validation demonstrated strong content validity, indicating its value as a practical resource for PWD and their care services.

## 1 Introduction

The global prevalence of dementia has exceeded 55 million cases, with 10 million new cases emerging annually—an estimate projected to ascend to 152 million by the year 2050 (1). Given that advanced age is recognized as the primary risk factor (2), the ongoing aging of the population virtually ensures a continuous increase in dementia cases across the globe. In parallel, national demographic data indicate a concurrent decline in family caregiver support for the older population, although the specific caregiver support indicators and their definitions differ across countries. In the United States, caregiver support ratios reported by Redfoot et al. declined from 7:1 in 2013 to an estimated 3:1 by 2050 (3) while national statistics from China show a corresponding reduction from 6:1 in 2016 to 4:1 by 2024 (4). Additionally, comparable shortages are present within formal care systems, which include a lack of geriatric specialists in America and a shortfall of 5.5 million eldercare personnel in China (5), collectively resulting in unsustainable pressures on global caregiving resources.

In addition to the growing demand for long-term care systems, caregivers of persons with dementia (PWD) are facing increasingly severe physical and psychological challenges. As a syndrome of cognitive dysfunction caused by brain damage or disease (1, 6), the progressively worsening memory impairment of PWD, thought disorders, and neuropsychiatric symptoms eventually render them dependent on caregivers for daily living (7). Studies indicate that most PWD primarily reside at home, receiving care and support from family members and other unpaid caregivers who lack professional knowledge and skills (8). Consequently, caregivers often endure prolonged and cumulative negative experiences including heightened stress, social isolation, frustration, and difficulties, along with the substantial emotional burden of caregiving (9). Notably, caregiving burden shows a positive correlation with unmet support needs (10). However, caregivers encounter multiple barriers when seeking support, including psychological factors (e.g., stigma and reluctance to seek help), difficulties in accessing information (11), as well as time and geographical constraints (12).

In response to the multiple needs of caregivers of PWD in terms of knowledge, skills, and psychological support, dementia carer education is considered an important strategy for enhancing caregiving ability and promoting caregiving quality (13). Systematic dementia carer education helps caregivers understand the pathological mechanisms of dementia and the patterns of behavioral changes, while equipping them with evidence-informed caregiving skills and communication methods, thereby improving caregiving behaviors and reducing neuropsychiatric symptoms in PWD (14, 15). Furthermore, dementia carer education can enhance caregivers’ self-efficacy and confidence, helping them manage stress and emotions more effectively (14). Among various forms of dementia carer education, informational manuals, as structured, low-cost, and reusable educational tools, have been widely applied in the fields of chronic diseases and long-term care. Their content typically covers disease knowledge, caregiving skills, psychological coping strategies, and resource access, helping caregivers systematically learn and continuously reference the material in daily practice (16, 17). Compared to one-time education or short-term training, manuals offer the advantage of being reusable, providing long-term guidance and psychological support across different stages of caregiving, thereby ensuring the continuous stimulation and support of caregiving behavior. Given the current situation of family caregiving for PWD in China, with older adult spouses and middle-aged family members as the primary caregivers, challenges such as inadequate digital literacy, limited access to information, and restricted professional support are prevalent (18). Paper-based manuals can effectively alleviate these challenges, as they can be promoted at low cost in community health service centers, grassroots clinics, neighborhood (village) committees, and long-term care institutions. Additionally, integrating these manuals with online platforms, such as WeChat public accounts, can broaden their reach and enable educational content to reach a wider audience.

Although relatively few studies have explicitly reported the development of dementia caregiving manuals, existing manualised interventions have primarily aimed to support caregivers in managing caregiving challenges and reducing psychological distress. For example, Kinnunen et al.’s Dementia Sleep Manual (19) provides structured guidance on understanding sleep in dementia, organising daily routines, responding to night-time challenging behaviors, and supporting caregivers’ own sleep. In contrast, Livingston et al.’s START programme (20) incorporates, in addition to dementia knowledge and behavioral management strategies, components such as cognitive and emotional regulation, acceptance, communication, relaxation, future planning, and engagement in pleasant activities. However, these positive-oriented components are predominantly incorporated to support burden reduction and are not positioned as a core focus in the development of manual content aimed at enhancing the positive aspects of caregiving.

In recent years, positive psychology has discovered that caregivers can still find benefits in stressful situations, leading to a sense of positive aspect of caregiving (21)—such as a sense of achievement, self-worth, and fulfillment—that caregivers experience during the caregiving process (22). These positive effects not only help caregivers cope with stress and reduce negative emotions but also enhance emotional support and a sense of security for PWD, improving their quality of life and neuropsychiatric symptoms (23). The perspective of positive psychology emphasizes cultivating and strengthening these positive factors, which have significant potential for improving caregiver well-being. A few scholars have begun attempting to combine traditional problem-solving strategies with the cultivation of the positive aspect of caregiving to help caregivers understand dementia caregiving from the perspective of positive psychology. For example, the World Health Organization’s iSupport program incorporates the positive aspect of caregiving as one of the key assessment indicators (24). Cheng et al. (25) developed the “Benefit Discovery Intervention” program, which not only includes practical caregiving skills but also enhances psychological resilience by guiding caregivers to discover positive meaning. Additionally, studies have shown that psychological interventions, such as caregiving skills, emotion management (26), rational emotive therapy (27), and community home visit services (28), can improve the positive aspect of caregiving for caregivers of PWD. These studies suggest that, alongside addressing practical issues, helping caregivers reconstruct the meaning of caregiving may be more sustainable and beneficial overall than interventions focusing on a single dimension. Therefore, the design of this manual not only focuses on alleviating caregiver burden but also emphasizes strategies to enhance caregivers’ positive aspect of caregiving, helping them discover positive meaning in caregiving, strengthen psychological resilience, and promote the sustainable development of long-term psychological well-being.

Therefore, this study aims to integrate problem-focused, emotion-focused, and meaning-focused coping strategies to provide a theory-based dementia carer education manual for caregivers of PWD. In addition to the paper-based manual, digital resources will also be provided through platforms such as WeChat public accounts, enabling more caregivers to access the manual.

## 2 Methods

### 2.1 Study design

**Figure 1:**
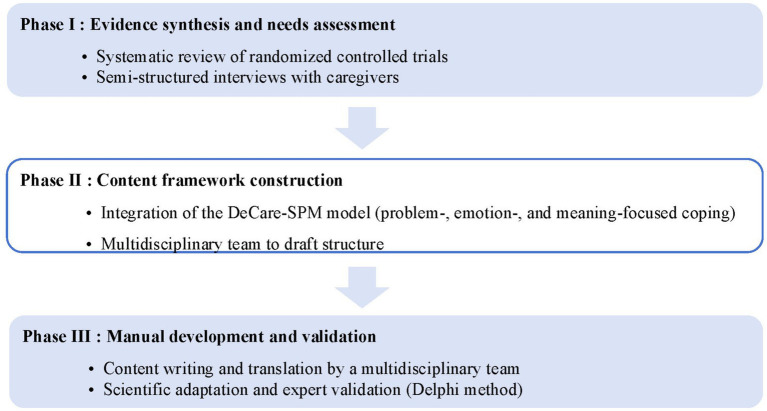
Methodological workflow of the study.

The study employed a multi-phase design comprising three primary stages: (I) Evidence synthesis and needs assessment, which included a systematic review of randomized controlled trials and semi-structured interviews with caregivers; (II) Content framework construction, involving a multidisciplinary team to draft the structure and integration of the DeCare-SPM model (encompassing problem-, emotion-, and meaning-focused coping strategies); and (III) Manual development and validation, where a multidisciplinary team conducted content writing and translation, followed by expert validation using the Delphi method. This structured approach ensured a comprehensive and rigorously validated outcome (see Figure 1).

### 2.2 Phase I: evidence synthesis and needs assessment

To inform the content of the caregiver education manual, Phase I combined evidence on interventions aimed at enhancing the positive aspects of caregiving with insights into the real-world needs and challenges of family caregivers.

First, a systematic review was conducted to identify interventions targeting the positive aspects of caregiving. Existing reviews have primarily focused on reducing caregiver burden and other negative outcomes, while systematic evaluations of interventions promoting the positive aspect remain limited. Relevant studies published in Chinese or English between 2001 and 2025 were identified, as the concept of the positive aspect of caregiving began to gain attention following the emergence of positive psychology in the late 20th century. Their types, content, delivery methods, and key characteristics were analyzed to extract evidence-based components for the manual. Inclusion criteria were as follows: (a) Study design and participants: Randomized controlled trials (RCTs) involving adult persons with dementia (≥18 years) living in community or home settings and their family caregivers (spouses, adult children, or other relatives); (b) Interventions: Any type of intervention targeting family caregivers, including caregiver-only or dyadic interventions involving both the caregiver and the PWD; (c) Outcome measures: Studies reporting positive aspect of caregiving among family caregivers of PWD. In this review, positive aspect of caregiving was defined as positive experiences or gains explicitly attributed to the caregiving role, including personal growth and the acquisition of caregiving-related knowledge and skills. Exclusion criteria were as follows: (a) Multiple publications from the same study: Only the most comprehensive publication was included, and other duplicates were excluded; (b) Studies involving care recipients without a confirmed dementia diagnosis; (c) Interventions involving pharmacological treatment for the PWD or non-pharmacological interventions targeting only the PWD; (d) Studies were excluded if outcome measures were not explicitly described or discussed as positive aspect of caregiving derived from the caregiving experience, including studies that reported outcomes such as self-efficacy, satisfaction, or well-being without explicitly attributing these outcomes to the caregiving experience; (e) Studies with unavailable full text or incomplete data, or published in languages other than Chinese or English.

**Figure 2:**
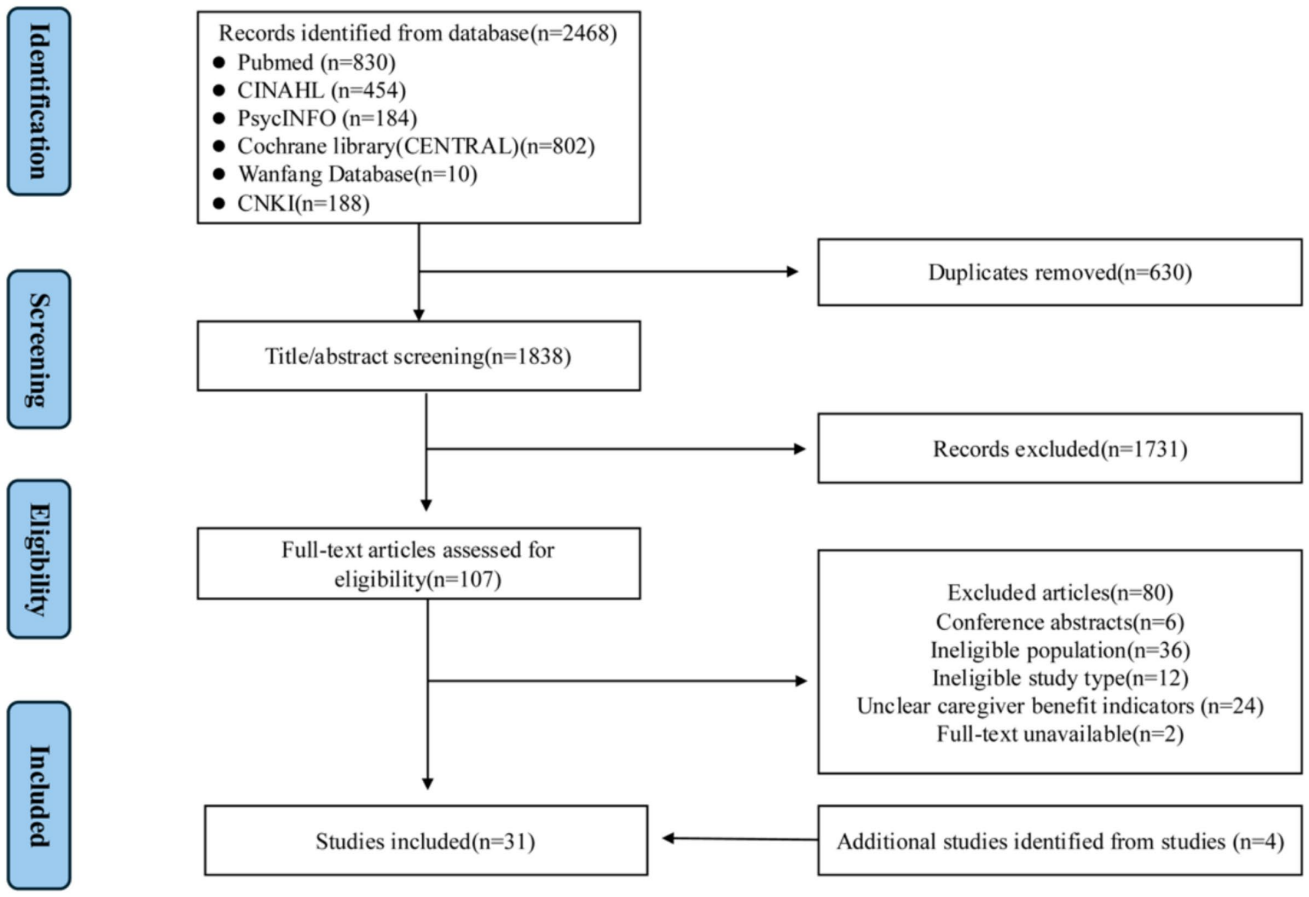
Flowchart of literature selection.

The study selection process is illustrated in Figure 2. Details of the search strategy are provided in Supplementary material A.

Second, the study team developed an interview guide based on the theoretical framework and clinical experience. Interviews with family caregivers of PWD were conducted following informed consent, and data collection continued until no substantially new themes were identified. Interviews explored several aspects of caregiving needs, including: (1) challenges encountered in daily care and emotional management, (2) perceived sources of stress and coping strategies, (3) expectations for educational support and available resources, and (4) perceived changes in personal growth or meaning during caregiving. Data were analyzed using inductive content analysis, with codes and themes generated from recurring patterns and concepts.

### 2.3 Phase II: content framework construction

Guided by the Dementia Caregiver Stress Process Model (DeCare-SPM), which was previously outlined in our published study protocol (29), this study established the framework for the manual. Three coping strategies were focused on: (a) problem-focused coping employs active behavioral strategies (e.g., time management, skill acquisition) to directly address and modify stress-inducing environmental factors; (b) emotion-focused coping utilizes affective regulation techniques (e.g., relaxation training, emotional articulation) to mitigate distressing emotional states arising from caregiving demands; and (c) meaning-focused coping facilitates adaptive cognitive restructuring (e.g., spiritual engagement, positive reappraisal) to promote psychological resilience and positive adjustment.

Building upon this framework, a multidisciplinary team developed the content framework of the manual by integrating findings from the literature review and qualitative interviews.

### 2.4 Phase III: manual development and validation

Based on the pre-established content indicator system, the multidisciplinary team systematically refined the content at all levels. Following the EM Guidelines (30), professional content was transformed into plain language while avoiding technical jargon. The visual design incorporated intuitive layouts with carefully selected illustrations and cartoons to create an approachable style. To ensure wide accessibility and practical utility, the manual was finalized for dissemination in two complementary formats: a printed booklet for offline reference and a digitally identical version deployed as a series of posts on a WeChat Official Account for online access and ease of sharing. Through iterative refinements, the manual achieved clear text, concise language, intuitive visuals, logical layouts, and user-friendly design. Based on expert panel discussion, specific content and design elements of the manual were reviewed and discussed item by item until consensus was reached. Subsequently, the researchers implemented the corresponding modifications to the manual’s content and design. The experts were then invited to evaluate the manual based on predefined indicators, assessing its importance and practicality using a 5-point Likert scale (1 = strongly disagree to 5 = strongly agree). Higher scores indicated greater importance and better comprehensibility. Expert evaluation data were imported into Microsoft Office Excel 7.0 for content validity analysis. The item-level content validity index (I-CVI) was calculated as the proportion of experts rating each item as either 4 or 5, and the scale-level content validity index (S-CVI/Ave) was obtained by averaging all I-CVI values. Items with an inter-expert agreement ≥0.8 were considered valid (31).

## 3 Results

### 3.1 Types and core components of interventions identified in the systematic review

The initial study identified 2468 potentially relevant articles, and after screening abstracts and full texts, 31 studies were ultimately included. The methodological quality assessment found that 8.1% of the studies had a high risk of blinding participants and personnel. The lowest risks were observed in random sequence generation (45.2%) and completeness of outcome data reporting (58.1%) (see Supplementary material B). The systematic review identified seven types of interventions for family caregivers of PWD through an inductive analysis of intervention content (32). The characteristics and elements of these interventions are summarized in Table 1 and include psychosocial interventions, psychoeducational interventions, web-based interventions, support groups, cognitive behavioral therapy (CBT), writing therapy, and behavioral activation. Among these, psychosocial and psychoeducational interventions were the most commonly reported. Content analysis revealed that psychosocial interventions were multi-component, typically integrating education, CBT techniques, and support groups. Both psychosocial and psychoeducational interventions shared core elements of disease knowledge education, caregiving skills training, and psychological support, with education and skills training particularly emphasized for addressing common caregiving challenges and stress. CBT, as a component of psychosocial interventions, targets cognitive restructuring, problem-solving, goal setting, and emotion regulation strategies—such as mindfulness, gratitude exercises, and positive reappraisal—and these strategies are essential for fostering positive caregiving experiences.

**Table 1:** The characteristics and elements of interventions.

Author(s)	Year	Country	Intervention type	Intervention content	Outcome effects
Kuo (33)	2016	China	Psychosocial Intervention	Patient/family assessment, Behavioral and Psychological Symptoms of Dementia management, community service referrals	②+/④+/⑥+
Gallagher-Thompson (34)	2010	USA	Psychosocial Intervention	Dementia education, stress management, communication skills, Behavioral and Psychological Symptoms of Dementia coping, resource acquisition, legal issues, end-of-life care	①+/④−
Hepburn (35)	2022	USA	Psychosocial Intervention	Tele-Savvy program (core knowledge, emotional coping, resources) + 6 mindfulness sessions	②+/③−/④−/⑤+
Weiping Zang (28)	2017	China	Psychosocial Intervention	Dementia knowledge, Activities of Daily Living care, rehabilitation nursing, home safety, self-care guidance	①+
Seike (36)	2021	Japan	Psychosocial Intervention	Core dementia knowledge, stress management, support-seeking skills	①−/⑤+
Tamura (37)	2023	Japan	Psychoeducational Intervention	/	①+/③ + ④−/⑤−/⑥+
Gossink (38)	2018	Netherlands	Psychoeducational Intervention	Social support, education, problem-solving, behavioral training	②−/③−/④−
Yoo (39)	2019	Korea	Psychoeducational Intervention	Day club activities, support group meetings, counseling, social events	①−/③+/⑤+/⑥−
Czaja (40)	2013	USA	Psychoeducational Intervention	Video calls are not only used for meetings but also offer resource guides, care tips, and educational video modules (updated monthly).	①+/④−
Cheng (25)	2016	China	Psychoeducational Intervention	Educational video modules (monthly updates), positive reappraisal, benefit-finding journaling	①+/③+/⑤+
Gonzalez (41)	2014	USA	CBT	Six modules: Use FOCUS (focus, optimism, creativity, understanding, solution) as the mnemonic phrase, covering problem description, goal setting, inspiring optimism, inspiring creative solutions, understanding patient preferences, implementing and evaluating solutions	①−/④−⑤−
Moskowitz (42)	2019	USA	iCBT	8 emotion regulation skills: positive event attention/utilization, gratitude, mindfulness, reappraisal, strength focus, goal-setting, kindness	①+/③−④+/⑤+
Fossey (43)	2021	UK	iCBT	Dementia awareness, self-knowledge, stress/emotion management, interpersonal skills, self-care, coping plans	②−/④−/⑤−
Teles (24)	2022	Portugal	Web-Based Intervention	WHO iSupport modules: dementia overview, caregiving roles, self-care, Activities of Daily Living management, Behavioral and Psychological Symptoms of Dementia coping	①−/③−/④+/⑤−/⑥+
Williams (44)	2019	USA	Web-Based Intervention	Behavior-tracking app with expert-customized interventions	②+/③−/⑤+
Beauchamp (45)	2005	USA	Web-Based Intervention	Becoming a caregiver, dealing with emotions and common difficulties, problem-solving skills and social support skills	①+/④+/⑤+
Gustafson (46)	2019	USA	Web-Based Intervention	Bluetooth/GPS tracking, motion sensors for safety alerts	②+/③+/④−/⑤-
Boots (47)	2018	Netherlands	Web-Based Intervention	Dementia education, Activities of Daily Living care, rehabilitation, home safety, self-care	②+/④−/⑤−/⑥+
Duggleby (48)	2018	Canada	Web-Based Intervention	5 modules: personal profile, expected changes, Frequently Asked Questions resources, health info, calendar	①−/⑥−
Hattink (49)	2015	Netherlands/UK	Web-Based Intervention	8 themes: dementia basics, daily living challenges, Behavioral and Psychological Symptoms of Dementia management, communication strategies, self-care	②+/③+/⑥+
Beentjes (50)	2020	Netherlands	Web-Based Intervention	FindMyApps database (self-management, social engagement, meaningful activities)	①−/②−/⑥−
Dröes (51)	2019	Netherlands	Web-Based Intervention	Daytime clubs, group training support meetings, regular consultations and social activities	②−/③−/⑥−
Núñez-Naveira (52)	2016	Spain	Web-Based Intervention	Provide information in five modules: cognitive decline, daily tasks, behavioral changes, social activities, and you as a caregiver	②−/⑤+
Moore (53)	2013	USA	Behavioral Activation	/	①−/④−
Xu (54)	2022	China	Behavioral Activation	/	①+/⑤+
Butcher (55)	2016	USA	Writing Therapy	/	①+/②−/⑤−
DeGregory (56)	2014	USA	Writing Therapy	/	①−/③−/④−
Fuju (57)	2021	Japan	Writing Therapy	/	②−/③+/⑤+
Winter (58)	2006	USA	Support Group	/	①−/③−/④−
Charlesworth (59)	2008	UK	Support Group	/	①−/④−/⑤−/⑥−
Laakkonen (60)	2016	Finland	Support Group	/	②−⑥−

① The positive aspect of caregiving; ② Caregiving competence; ③ Caregiving burden; ④ Anxiety; ⑤ Depression; ⑥ Quality of life; “+” Significant effect; “−” Non-significant effect, the outcome effects of the first follow-up time were included under multiple follow-up times.

### 3.2 Barriers and support needs in dementia care for family caregivers of PWD

In-depth qualitative interviews were conducted with 8 family caregivers (3 males, 5 females) lasting 23–47 min each, until data saturation was reached. Caregivers were aged 49–72 years and had educational levels ranging from primary school to bachelor’s degree. Caregiving experience varied from 6 months to over 4 years, and most participants were adult children (6/8), with the remainder being spouses (2/8). Through qualitative analysis, we found that family caregivers of PWD face multiple barriers in accessing care support. The barriers for family caregivers of PWD seeking support included socio-geographical isolation and ineffective help in terms of social support network accessibility; fear of being a burden and stigma associated with the illness in terms of reciprocity; and lack of trust in external caregiving services, which may lead to reluctance to seek help outside the family, as well as role limitations, where caregivers feel that seeking external help might imply a failure to fulfill familial duties. Family caregivers perceived economic and practical support, emotional and psychological support, but noticed limited formal support from healthcare providers. Caregivers expressed needs for training in dementia care, especially in behavioral and psychological symptom management, emotional and psychological support, and early stress management strategies. These needs aligned closely with the core elements identified in the systematic review, particularly in problem-solving, emotional regulation, and coping skills. Key themes and representative quotations from the qualitative interviews are presented in Supplementary material C.

### 3.3 Content framework of the manual

A multidisciplinary team was formed, comprising geriatricians, geriatric nurses, mental health specialists, social workers, psychologists, and dementia care professionals (two representatives per discipline). The intervention elements identified in the systematic review were mapped onto the real-world caregiving challenges reported in the qualitative study. Problem-solving strategies corresponded to daily caregiving pressures; CBT and emotion regulation strategies were linked to emotional management challenges; and positive psychology strategies (e.g., meaning-focused coping, cognitive reappraisal) were incorporated to enhance psychological resilience and positive caregiving experiences. Table 2 presents how each manual module integrates evidence-based intervention elements with caregiver-identified needs. Based on these mappings, the multidisciplinary team iteratively discussed and refined the content. The content system of the manual was initially constructed, encompassing primary, secondary, and tertiary indicators to guide module development. Table 3 presents the finalized content framework of the manual.

**Table 2:** Mapping intervention elements and caregiver needs onto manual modules.

Systematic elements	Caregiver needs	Module
Education CBT problem-solving strategies	Daily caregiving challenges Behavioral symptom management needs	Problem-based coping
CBT emotion regulation techniques Mindfulness Gratitude exercises	Emotional stress management needs Psychological support needs	Emotion-based coping
Positive psychology strategies Cognitive reappraisal	Personal growth Meaning-making needs	Meaning-based Coping

**Table 3:** Content framework of the health education manual for caregivers of PWD.

Primary indicators	Secondary indicators	Tertiary indicators
1. Problem-based coping	1.1 Understanding dementia	1.1.1 What is dementia 1.1.2 What causes dementia 1.1.3 Progression of dementia 1.1.4 How to intervene and alleviate
	1.2 Daily care	1.2.1 How to communicate with dementia 1.2.2 How to create a dementia-friendly home 1.2.3 Diet for dementia 1.2.4 Urination and defecation 1.2.5 Take a bath
	1.3 Mental and behavioral symptoms	1.3.1 Delusion/Hallucination 1.3.2 Aggressive behavior 1.3.3 Agitation behavior 1.3.4 Anxiety/Depression/Apathy 1.3.5 Loss of control of behavior 1.3.6 Wander/linger/get lost 1.3.7 Nocturnal behavior 1.3.8 Changes in eating behavior
2. Emotion-based coping	2.1 Identifying anxiety, depression, and relaxation techniques	2.1.1 Identification of negative feelings 2.1.2 Learn how to relax
	2.2 Mindfulness training	2.2.1 The concept and basic functions of mindfulness 2.2.2 Mindful breathing 2.2.3 Mindfulness stretching
	2.3 Behavior activation	2.3.1 Cognitive behavioral therapy 2.3.2 Pleasant activities
3. Meaning-based coping	3.1 Self-management	3.1.1 Self-care 3.1.2 Change your mind
	3.2 Benefit finding	3.2.1 The concept of sense of meaning 3.2.2 Source of the sense of meaning 3.2.3 Performance of the sense of meaning
	3.3 Positive appraisal	3.3.1 Goal Setting 3.3.2 Seeking Help

### 3.4 Manual design and content validation results

Following discussion of the multidisciplinary team, the overall design of the manual adopted orange as the primary color, with text in 14 pt. Times New Roman font and 1.5 line spacing to ensure readability. The content was organized with a focus on conceptual explanations and coping strategies, supplemented with illustrative graphics. Key information was highlighted using color blocks and bulleted lists, while maintaining a clean and clear layout. The presentation format of the manual was optimized after multiple rounds of team feedback, ensuring a balance between scientific rigor and usability. The manual was structured into three main sections, with the content presentation format for each section detailed in Table 4.

**Table 4:** Content presentation format of the manual.

Section name and core content	Presentation format
Problem-focused coping Dementia basics, daily care techniques, behavioral symptom management	Conceptual diagrams Scenario-based comics Step-by-step flowcharts Video QR codes
Emotion-focused coping Stress adaptation, mindfulness exercises, behavioral activation	Self-assessment scales Training illustrations Audiovisual guidance QR codes Training log sheets
Meaning-focused coping Stress adaptation, mindfulness exercises, behavioral activation	Care checklists Progress trackers Resource QR codes

Following the development of the initial manual draft by the multidisciplinary team (MDT), the expert panel was consulted to assess and provide feedback. The study involved 11 experts, including mental health professionals (*n* = 3), geriatric nursing specialists (*n* = 3), community health specialists (*n* = 1), geriatricians (*n* = 1), frontline dementia care workers (*n* = 2), and social workers (*n* = 1). The panel consisted of 9 female participants (81.8%) with an average of 14 years of professional experience. Detailed characteristics of the expert panel are presented in Table 5.

**Table 5:** Characteristics of the experts (*n* = 11).

Variable	*n*	%
Gender		
Male	2	18.2
Female	9	81.8
Level of education		
Bachelor’s degree	4	36.4
Master’s degree	3	27.3
Doctoral degree	4	36.4
Professional experience (years)		
≦5	2	18.2
5–10	3	27.3
≧10	6	54.5
Professional disciplines		
Mental/Psychiatric Nursing	3	27.3
Geriatric Nursing	3	27.3
Geriatrics	1	9.1
Dementia Care	3	27.3
Social Work	1	9.1

During the expert panel discussion, several modifications were suggested. These included simplifying phrasing for clarity (e.g., changing “Different behavioral changes occur at different time points in the early and middle stages of dementia” to “Different behavioral changes occur at different time points in dementia”), reorganizing sections to improve logical flow (such as separating “Other Risk Factors” into “Risk Factors for Dementia”), and enhancing the visual presentation by adding color blocks, diagrams, and annotations. Experts also emphasized the importance of combining pharmacological and non-pharmacological treatments in delaying disease progression. Detailed expert feedback and the final version of the manual, based on these revisions, can be found in Supplementary material D.

As shown in Table 6, expert evaluations indicated that all sections of the manual achieved Content Validity Index (CVI) scores ranging from 0.81 to 1.00 for both relevance and comprehensibility. With CVIs ≥ 0.80 for both dimensions, the manual’s content was considered acceptable.

**Table 6:** Importance and understandability of the content of each subsection of the manual.

Section	Importance	Understandability
	CVI	CVI
Part 1: understanding the person you care for		
What is dementia	1.00	1.00
What causes dementia	0.91	0.91
Progression of dementia	1.00	0.82
How to intervene and alleviate	1.00	0.82
Delusion/hallucination	1.00	0.91
Aggressive behavior	1.00	1.00
Agitation behavior	1.00	0.91
Anxiety/depression/apathy	1.00	0.91
Loss of control of behavior	1.00	1.00
Wander/linger/get lost	1.00	1.00
Nocturnal behavior	1.00	0.91
Changes in eating behavior	1.00	0.91
Part 2: managing daily caregiving challenges		
Communicating with PWD	1.00	0.91
Creating a dementia-friendly home	1.00	0.91
Nutrition & eating support	1.00	1.00
Toileting assistance	1.00	0.91
Bathing support	0.82	0.91
Part 3: adapting to the caregiver role		
Identification of negative feelings	1.00	0.91
Learn how to relax yourself	1.00	0.91
The concept and basic functions of mindfulness	0.82	0.91
Mindful breathing	0.91	0.91
Mindfulness stretching	0.91	0.91
Cognitive behavioral therapy	1.00	0.91
Pleasant activities	1.00	0.91
Part 4: finding meaning in the caregiving journey		
Self-care	1.00	0.91
Change your mind	1.00	0.91
The concept of sense of meaning	1.00	1.00
Source of the sense of meaning	1.00	0.91
Performance of the sense of meaning	0.91	1.00
Goal setting	0.91	0.91
Seeking help	1.00	0.91
CVI-EI	0.97	0.92

## 4 Discussion and conclusion

### 4.1 Discussion

This study, based on the DeCare-SPM framework, integrated findings from literature reviews and qualitative needs assessments to develop a set of core evidence-based educational content for caregivers of PWD. To enhance both the reach and usability of the educational content, a unified content approach with dual delivery formats was adopted, presenting the materials simultaneously in a paper-based manual and through WeChat public accounts, thereby accommodating caregivers with varying levels of digital literacy and usage habits.

Currently, health education for caregivers of PWD has taken diverse forms, including digital interventions [e.g., mobile applications (45), online courses (48)], group training (61), individualized counseling, and paper-based educational materials (62). Digital tools, while facilitating information dissemination, may pose challenges for older caregivers with limited technical skills (63). Conversely, traditional paper-based materials, while offering advantages such as low cost and long-term preservation, have certain limitations in terms of dissemination reach and coverage (64). Therefore, combining paper-based and digital formats allows the core knowledge to be more accessible while expanding the reach and sustainability of the educational content.

The selection of a paper-based manual as a primary delivery format was guided by three considerations. First, paper-based materials align more closely with the usage habits and capabilities of the target population. The primary audience in this study comprises family caregivers of PWD, particularly older spouses (65), who often have lower educational levels and may encounter difficulties using digital devices (66). Previous studies indicate that individuals with lower educational attainment and socioeconomic status are less able to effectively utilize digital tools to access health information, thereby limiting the use of digital health services (67). In this context, paper-based manuals offer a practical advantage, as they can be accessed without electronic devices, lowering learning barriers, enhancing information accessibility, and supporting caregivers in consolidating knowledge through repeated use-features that are particularly valuable in resource-limited community settings (68).

Meanwhile, digital tools, such as content dissemination via WeChat, enable convenient reach to younger family members and caregivers familiar with digital platforms, leveraging social sharing features to broaden dissemination (69, 70). This approach not only extends the reach of the manual but also enhances the efficiency and flexibility of educational resource delivery, offering caregivers multiple pathways for learning.

Furthermore, the manual demonstrates strong applicability and scalability. It can be distributed and collected through community health service centers, primary healthcare institutions, long-term care facilities, and government initiatives (e.g., dementia-friendly communities), and can be periodically issued and revisited during home visits, caregiver classes, and outpatient education sessions.

In the design process, we fully referenced existing studies and theoretical frameworks to ensure the manual’s scientific and practical validity. For example, in terms of language style, the manual incorporates Shaari’s conversational expression and terminology annotation (71), ensuring that the content is both easy to understand and professionally appropriate. Furthermore, the use of vivid illustrations and cartoon characters enhances the visual effect, significantly improving readability and caregiver comprehension (72, 73). To address the issue of excessive terminology identified in previous studies (74), this manual employs an information layering design that combines core points with operational details, ensuring clarity and brevity. Moreover, drawing from Rong’s findings on the use of comics to enhance readability (75), the manual features unique character images, presenting key content through combinations of text, situational simulations, and videos accessible via QR codes. For example, simulated scenes of various neuropsychiatric symptoms of dementia, home environment design diagrams, and dietary examples are presented. The use of the more engaging second-person “you” avoids the third-person terms “he, or the patient” and fosters empathy, while typical case analyses promote knowledge transfer (76).

One key advantage of this manual is that it directly responds to unmet needs reported by caregivers. Qualitative results indicated that caregivers often experience significant emotional and operational pressure when managing the behavioral and neuropsychiatric symptoms, particularly when dealing with symptoms like agitation, hallucinations, and sleep disturbances. This finding is consistent with existing studies, which identify neuropsychiatric symptoms as one of the main sources of caregiver stress (77). Therefore, the manual provides targeted strategies and scenario-based illustrations to help caregivers understand and cope with neuropsychiatric symptoms through non-pharmacological means.

In addition to the common “problem-focused” content, this manual includes the dimension of “meaning-focused coping,” which, although theoretically recognized, is often overlooked in existing caregiver education materials (78). Studies have indicated that the positive aspect of caregiving—that is, reconstructing personal experiences, exploring one’s values, and finding life goals through caregiving—can significantly enhance the caregiving experience (79). Although a unified definition of this concept remains elusive, and optimal interventions are not yet established, studies have shown that helping caregivers reframe stressors and interpret them from a more positive perspective can foster better emotional responses and improved caregiving outcomes. For instance, Cheng’s psychoeducational program (25) guided caregivers in emotional reappraisal, helping them identify and transform stressors to achieve a more positive psychological experience. This approach aligns with the content design of the manual. As a result, this manual not only aims to alleviate caregiver stress but also emphasizes promoting their resilience and psychological growth. Unlike traditional manuals that focus on knowledge transmission and stress relief, this manual guides caregivers in finding personal meaning in their caregiving experiences, encouraging them to redefine challenges in caregiving and thus achieve positive psychological adaptation.

The qualitative interview results also revealed that caregivers frequently face stigma during caregiving. Consequently, this manual places particular emphasis on a person-centered care philosophy, advocating for mutual respect and understanding between caregivers and care recipients. In terms of language, the manual adopts a friendly tone, avoiding terms that may trigger negative emotions or discrimination, thereby creating a more respectful and understanding caregiving environment.

This study has several limitations. First, the expert panel was largely recruited from a single geographic region, potentially constraining regional representativeness. Nevertheless, the panelists possessed substantial clinical and research expertise in dementia care support, which lends authority and credibility to the findings. Second, the study did not include a pilot application of the manual to assess its impact on caregivers’ knowledge, skills, and health-related outcomes, thereby limiting the evaluation of its practical effectiveness. Third, the study did not include direct input from caregivers beyond the qualitative interviews. Caregivers could participate as part of the multidisciplinary team or in aspects of the design and acceptability assessment. Future investigations should prioritize rigorous evaluation studies to examine the manual’s usability, effectiveness, and cost-effectiveness among PWD and their caregivers. Additionally, future research should incorporate caregiver involvement to further assess the manual’s relevance and acceptability.

### 4.2 Conclusion

This study developed a theory- and evidence-based health education manual for caregivers of PWD, integrating problem-focused, emotion-focused, and meaning-focused coping strategies to address their practical needs. The manual provides structured, accessible guidance to support caregivers in managing cognitive, emotional, and behavioral challenges in daily care. While it does not replace formal services, it serves as a complementary resource that can enhance caregiving capacity and be applied flexibly in home and community settings, including both paper-based and digital formats to broaden accessibility.

## Data Availability

The original contributions presented in the study are included in the article/Supplementary material, further inquiries can be directed to the corresponding author.
