# Supplementary material for: Development and content evaluation of a multidimensional coping model-based health education manual for dementia care support
Source: Front Public Health. 2026 Mar 16;14:1750034. doi: 10.3389/fpubh.2026.1750034 (PMC13033505; doi:10.3389/fpubh.2026.1750034)
Supplement: Supplementary file 1 [file Table_1.DOCX]

Supplementary Material

**(1)Supplementary Material A**

Search strategy in the PubMed

| **Steps** | **Search Results** |
| --- | --- |
| #1 | Search: caregivers[MeSH Terms] |
| #2 | Search family[MeSH Terms] |
| #3 | Search: (((carer*[Title/Abstract]) OR (caring[Title/Abstract])) OR (caretaker*[Title/Abstract])) OR (caregiver*[Title/Abstract]) |
| #4 | #1 OR #2 OR #3 |
| #5 | Search Dementia[MeSH Terms] |
| #6 | Search: (dement*[Title/Abstract]) OR (alzheimer*[Title/Abstract]) |
| #7 | #5 OR #6 |
| #8 | Search Psychology, Positive[MeSH Terms] |
| #9 | Search: (((((((((positive aspect[Title/Abstract]) OR (positive experience[Title/Abstract])) OR (positive perceptions[Title/Abstract])) OR (positive appraisal[Title/Abstract])) OR (reward*[Title/Abstract])) OR (gain*[Title/Abstract]))) OR (benefit*[Title/Abstract])) OR (meaning*[Title/Abstract])) OR (hope[Title/Abstract]) |
| #10 | #8 OR #9 |
| #11 | Search Controlled Clinical Trial[Publication Type] |
| #12 | Search (random*[Title/Abstract]) OR (trial*[Title/Abstract]) |
| #13 | #11 OR #12 |
| #14 | Search drug therapy[MeSH Terms] |
| #15 | Search: ((pharmacotherap*[Title/Abstract]) OR (chemotherap*[Title/Abstract])) OR (drug[Title/Abstract]) |
| #16 | #14 OR #15 |
| #17 | #4 AND #7 AND #10 AND #13 NOT #16 |
| #18 | (English[Language]) AND #4 AND #7 AND #10 AND #13 NOT #16 AND (“2000/01/01"[Date - Publication]: 2025/07/31"[Date - Publication]), 740 results for search |

**(2)Supplementary Material B**

Methodological quality assessment of included RCT studies

| Authors | Random sequence generation (selection bias) | Allocation concealment (selection bias) | Blinding of participants and personnel (performance bias) | Blinding of outcome assessment (detection bias) | Incomplete outcome data (attrition bias) | Selective reporting (reporting bias) | Others |
| --- | --- | --- | --- | --- | --- | --- | --- |
| Winter (57) | – | ? | + | ? | ? | ? | ? |
| Charlesworth (58) | – | – | + | ? | – | – | ? |
| Gallagher-Thompson (45) | ? | ? | ? | ? | – | ? | ? |
| Czaja (39) | ? | ? | + | – | – | ? | ? |
| DeGregory (39) | – | ? | + | ? | – | ? | ? |
| Gonzalez (40) | ? | ? | + | – | + | ? | ? |
| Butcher (54) | ? | ? | + | + | – | ? | ? |
| Cheng (24) | – | ? | – | + | – | ? | ? |
| Kuo (31) | – | ? | + | + | – | ? | ? |
| Boots (46) | – | ? | + | – | – | ? | ? |
| Duggleby (47) | + | + | + | + | + | + | + |
| Dröes (50) | + | ? | ? | ? | + | + | – |
| Gustafson (45) | ? | ? | ? | ? | + | + | – |
| Moskowitz (41) | – | ? | + | ? | – | – | ? |
| Beentjes (49) | ? | ? | + | + | – | ? | ? |
| Fossey (42) | – | ? | + | ? | – | ? | ? |
| Fuju (56) | – | ? | + | – | – | ? | ? |
| Hepburn (33) | – | ? | + | – | – | ? | ? |
| Teles (26) | + | + | + | – | + | + | + |
| Williams (43) | + | ? | ? | ? | + | + | + |
| Beauchamp (44) | ? | ? | ? | ? | – | ? | ? |
| Núñez-Naveira (51) | + | ? | ? | + | + | + | + |
| Laakkonen (59) | + | + | – | + | + | – | + |
| Yoo (38) | + | + | + | + | + | + | + |
| Moore (52) | – | ? | + | – | – | ? | ? |
| Gossink (37) | ? | ? | ? | ? | ? | ? | – |
| Hattink (48) | + | + | – | + | + | + | + |
| Seike (35) | – | ? | ? | – | + | ? | ? |
| Xu (53) | – | – | ? | ? | – | ? | ? |
| Tamura (36) | – | ? | + | + | – | ? | – |
| Zang Weiping (34) | ? | ? | ? | + | – | ? | – |
| + Low Risk | 25.8% | 16.1% | 58.1% | 35.5% | 35.5% | 25.8% | 22.6% |
| – High Risk | 45.2% | 6.5% | 9.7% | 25.8% | 58.1% | 9.7% | 16.1% |
| ? Unclear Risk | 29.0% | 77.4% | 32.3% | 38.7% | 6.5% | 64.5% | 61.3% |

**(3)Supplementary Material C**

Key Themes and Representative Quotations from Qualitative Interviews

| **Main Theme** | **Sub-theme** | **Brief description** | **Representative quotation(s)** |
| --- | --- | --- | --- |
| **Accessibility** | Social–geographical isolation | Reduced access to social interaction and healthcare support due to geographic distance and weakened neighbourhood connections | “Unlike before, neighbours no longer visit each other. I don’t even know the people living next door.” (C3)  “We live in a remote rural area, and it takes two to three hours by car to reach the nearest hospital. It’s very inconvenient.” (C4) |
|  | Unhelpful support | Previous experiences of ineffective support discouraged caregivers from seeking further help | “They don’t really understand what dementia is, so asking them doesn’t help. I didn’t know much myself before either.” (C1) |
|  | Digital competence | Online platforms enabled access to caregiving information and peer support | “There are many learning resources online. I follow a public WeChat account that shares information about dementia, and it’s quite helpful.” (C3) |
| **Reciprocity** | Fear of burdening others | Caregivers avoided seeking help to avoid troubling family members or friends | “Other people are not obliged to help you for free. It’s all about personal favours, so I try not to trouble others.” (C5) |
|  | Stigma | Dementia-related stigma limited disclosure and social engagement | “If people know someone has dementia, they start talking and judging. That’s why I didn’t tell others at the beginning.” (C8) |
|  | Social participation | Participation in organised activities facilitated information exchange and peer connection | “I met some social workers through activities organised by the university or hospital. I also met other caregivers there, and sometimes we can share information.” (C2) |
| **Trust** | Lack of trust | Distrust in external care services reduced willingness to seek formal support | “I don’t want others to suffer. You often see news reports about older people being abused.” (C5) |
|  | Role-based responsibility | A strong sense of familial responsibility constrained help-seeking behaviour | “As a child, caring for one’s parents is a family responsibility. Asking others for help feels like I haven’t fulfilled my duty.” (C1) |
|  | Close relationships | Trust in close family members or friends facilitated emotional disclosure and support seeking | “I only talk about these difficulties and seek help from relatives or friends I am very close to.” (C7) |

**(4)Supplementary Material D**

Detailed Expert Feedback

Expert 1 suggested simplifying some phrasing, such as changing "Different behavioral changes occur at different time points in the early and middle stages of dementia" to "Different behavioral changes occur at different time points in dementia." This suggestion was accepted, and the phrasing has been simplified for greater clarity.

Expert 4 recommended bolding the "20" in "20%-30%" in the section on vascular dementia to make the data more prominent. This suggestion was accepted, and the "20" has been bolded.

Expert 5 proposed separating "Other Risk Factors" into a distinct section titled "Risk Factors for Dementia." This was accepted, and the content structure has been adjusted for better logical flow.

Expert 6 pointed out the need to optimize the disease progression description, emphasizing that "pharmacological and non-pharmacological combined treatments can delay disease progression" and should be repeatedly highlighted. This suggestion was accepted, and the importance of combined treatments has been emphasized throughout the relevant sections.

Expert 9 recommended optimizing the mindfulness section by adopting a "group training" approach and encouraging "family member participation." This was accepted, and the mindfulness section has been revised to include more actionable group training formats with a focus on family support.

Expert 1 suggested using color blocks, diagrams, and annotations to highlight key information and enhance visual impact. This suggestion was accepted, and color blocks and diagram annotations have been added to the manual to improve the visual presentation of key information.

Expert 10 recommended optimizing the manual's layout by adjusting font sizes and formatting to make the content more readable and enhance usability. This suggestion was accepted, and the manual's layout has been adjusted to better suit caregivers' reading habits.

Sample pages of the manual

| 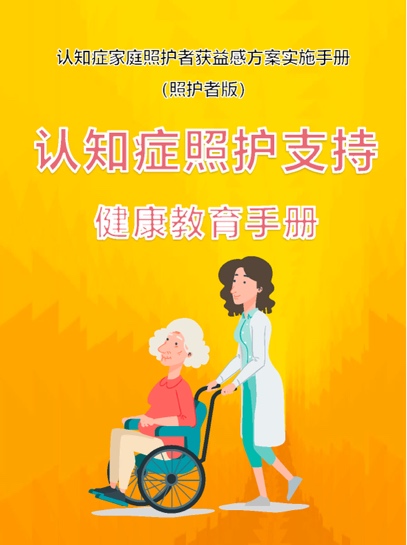 | 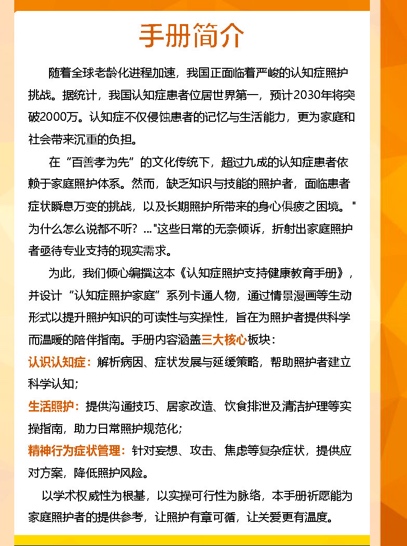 | 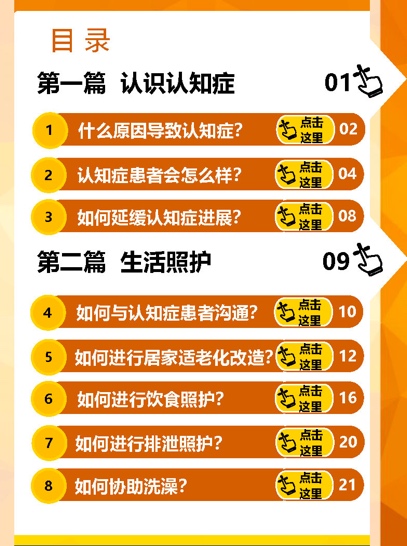 |
| --- | --- | --- |
| 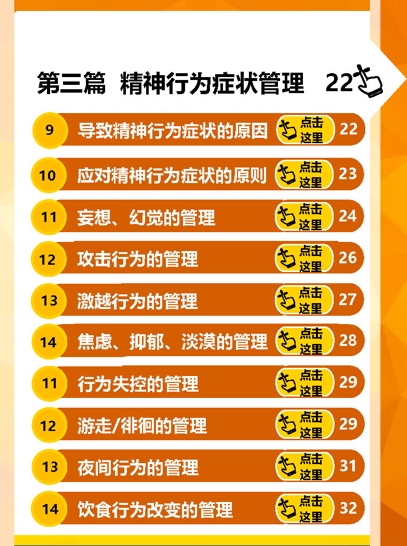 | 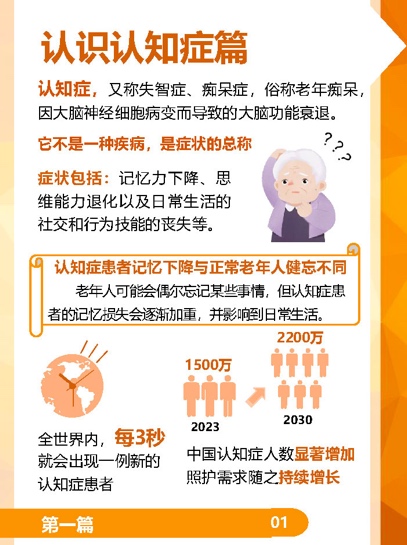 | 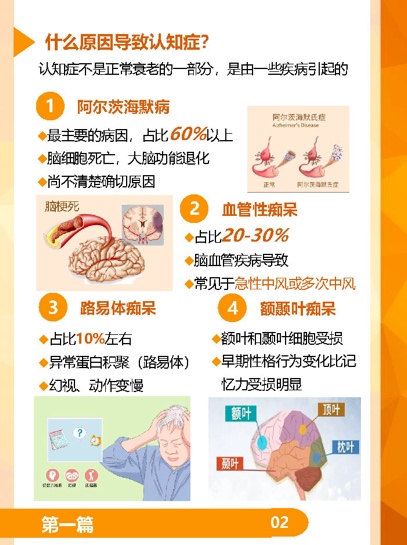 |
| 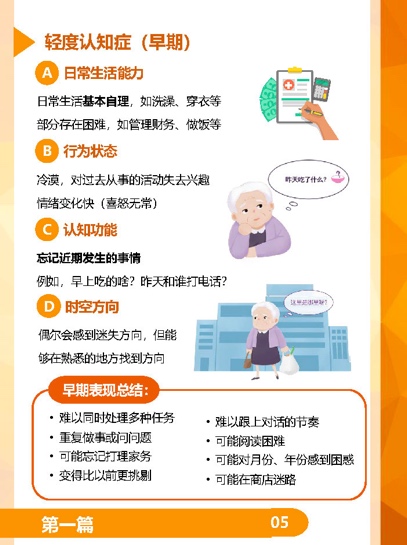 | 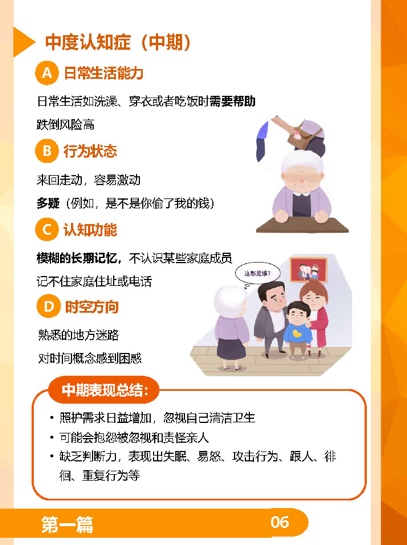 | 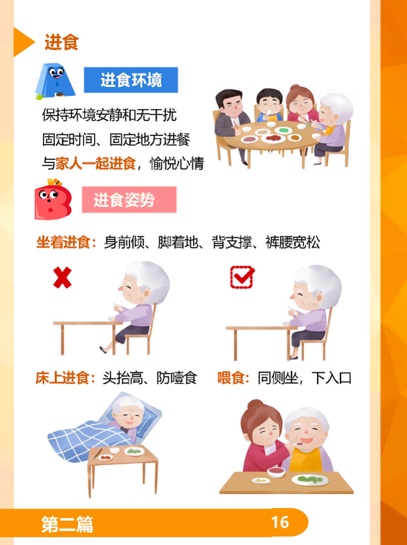 |
